# Integrated next-generation sequencing and comparative transcriptomic analysis of leaves provides novel insights into the ethylene pathway of *Chrysanthemum morifolium* in response to a Chinese isolate of chrysanthemum virus B

**DOI:** 10.1186/s12985-022-01890-3

**Published:** 2022-11-11

**Authors:** Xueting Zhong, Lianlian Yang, Jiapeng Li, Zhaoyang Tang, Choufei Wu, Liqin Zhang, Xueping Zhou, Yaqin Wang, Zhanqi Wang

**Affiliations:** 1 Key Laboratory of Vector Biology and Pathogen Control of Zhejiang Province, College of Life Sciences, Huzhou University, Huzhou, 313000 China; 2 State Key Laboratory of Rice Biology, Institute of Biotechnology, Zhejiang University, Hangzhou, 310058 China; 3 Key Laboratory for Biology of Plant Diseases and Insect Pests, Institute of Plant Protection, Chinese Academy of Agricultural Sciences, Beijing, 100193 China

**Keywords:** *Chrysanthemum morifolium*, Chrysanthemum virus B, Transcriptome, Ethylene pathway, Defense response

## Abstract

**Background:**

Chrysanthemum virus B (CVB), a key member of the genus *Carlavirus*, family *Betaflexiviridae*, causes severe viral diseases in chrysanthemum (*Chrysanthemum morifolium*) plants worldwide. However, information on the mechanisms underlying the response of chrysanthemum plants to CVB is scant.

**Methods:**

Here, an integrated next-generation sequencing and comparative transcriptomic analysis of chrysanthemum leaves was conducted to explore the molecular response mechanisms of plants to a Chinese isolate of CVB (CVB-CN) at the molecular level.

**Results:**

In total, 4934 significant differentially expressed genes (SDEGs) were identified to respond to CVB-CN, of which 4097 were upregulated and 837 were downregulated. Gene ontology and functional classification showed that the majority of upregulated SDEGs were categorized into gene cohorts involved in plant hormone signal transduction, phenylpropanoid and flavonoid biosynthesis, and ribosome metabolism. Enrichment analysis demonstrated that ethylene pathway-related genes were significantly upregulated following CVB-CN infection, indicating a strong promotion of ethylene biosynthesis and signaling. Furthermore, disruption of the ethylene pathway in *Nicotiana benthamiana*, a model plant, using virus-induced gene silencing technology rendered them more susceptible to cysteine-rich protein of CVB-CN induced hypersensitive response, suggesting a crucial role of this pathway in response to CVB-CN infection.

**Conclusion:**

This study provides evidence that ethylene pathway has an essential role of plant in response to CVB and offers valuable insights into the defense mechanisms of chrysanthemum against *Carlavirus*.

**Supplementary Information:**

The online version contains supplementary material available at 10.1186/s12985-022-01890-3.

## Background

Chrysanthemum (*Chrysanthemum morifolium*) is among the most economically important floricultural crops in *Asteraceae* worldwide [1, 2]. Chrysanthemum flowers are widely used in ornamentation, food, and tea owing to their high ornamental or edible value [3]. In China, chrysanthemum flowers have been used in traditional medicine considering their antioxidative, anti-inflammatory, antitumor, and hypolipidemic effects [4, 5]. Chrysanthemum plants are susceptible to virus invasion during cultivation, especially when grown on a large scale [6]. Virus-like symptoms such as foliar mosaic, stunting, and flower variegations, are commonly observed in chrysanthemum [6, 7]. To date, more than 20 viruses and viroids are reported to infect chrysanthemum; in China, the most problematic of which are chrysanthemum virus B (CVB), chrysanthemum virus R (CVR), cucumber mosaic virus (CMV), tomato aspermy virus (TAV), tobacco mosaic virus (TMV), potato virus Y (PVY), chrysanthemum stunt viroid (CSVd), and chrysanthemum chlorotic mottle viroid (CChMVd) [6, 8, 9].

CVB, which is transmitted by aphids and/or sap inoculation, is a major pathogen of chrysanthemum worldwide and causes symptoms ranging from leaf mottling to severe mosaic or flower malformations [10, 11]. It is a single-stranded positive RNA virus that belongs to the genus *Carlavirus* within the family *Betaflexiviridae*, and has a filamentous particle that is approximately 680 × 12 nm in size [11, 12]. The genome of CVB is approximately 9.0 kb, with a with a 5′-cap structure and a 3′-poly(A) tail, and encompasses six open reading frames (ORFs) [11, 13]. ORF1 encodes a large protein implicated in viral replication, ORF2 to ORF4 encode triple gene block (TGB) proteins that are implicated in viral movement, ORF5 encodes a coat protein (CP), and ORF6 encodes a 12 kD cysteine-rich protein (CRP), whose zinc finger domain can bind single-stranded and double-stranded nucleotides in vitro [14]. The heterologous expression of CRP in *Nicotiana tabacum* cv. Samsun or *Nicotiana benthamiana* seedlings using a potato virus X (PVX) vector could induce a hypersensitive response [14, 15]. Moreover, CRP can also function as a plant transcription factor and regulates cell and proliferation size in tobacco species [16]. Recently, CRP was demonstrated to be a symptom determinant [17] and a viral suppressor of RNA silencing during viral infection [18].

To date, 10 full-length genome sequences of CVB isolates from different regions and countries have been obtained. Of these, the full-length genome sequence of Japanese isolate (CVB-S) was the first to be obtained [11]. Subsequently, an infectious cDNA clone of CVB-S, pCVB, was constructed; however, no visible symptoms were observed in chrysanthemum seedlings infiltrated with pCVB [19]. In 2012, complete genome sequences of CVB-TN, CVB-PB, CVB-UP, and CVB-UK isolates from India were reported [13]. Sequence analysis showed that all the four Indian isolates were similar to each other and had a genome organization quite similar to that of CVB-S, with which they shared 70–73% sequence identity at the genome level [13]. More recently, using next-generation sequencing (NGS) technology, complete genome sequences of two Chinese isolates (CVB-HZ1 and CVB-HZ2) and three Russian isolates (CVB-GS1, CVB-GS2 and CVB-FY) were obtained from *Gynura japonica* and chrysanthemum, respectively [20, 21]. These genome sequences of CVB isolates from different hosts and countries are valuable and diverse materials for the study of pathogenicity mechanisms and functional genes of *Carlavirus*.

In recent years, although substantial research on genome characterization and pathogenesis of CVB has been conducted, studies on the mechanisms underlying the response of host plants to CVB are scarce. In this study, an integrated NGS and comparative transcriptomic analysis of chrysanthemum leaves was carried out to elucidate the mechanisms underlying the response of plants against CVB-CN infection at the molecular level. These findings improve our understanding of the critical aspects of the molecular mechanisms underlying the defense responses of chrysanthemum to *Carlavirus*.

## Methods

### Plant materials and sample collection

During a field survey on October 13, 2018, 12 chrysanthemum plants exhibiting severe leaf mottling and moderate vein clearing were collected from five points in a chrysanthemum garden of Institute of Agricultural Sciences of Tongxiang, Zhejiang Province, China, the distance between each ranged from 200 to 1000 m more than. The chrysanthemum seedlings were growed by gardeners on April 10, 2018. Whole plants were dug out and put into sampling bags, and their roots were kept moist during transport to the laboratory. The infected leaves were cut into pieces, weighing 100 mg each; nine replicates for each plant were collected. Healthy chrysanthemum plants served as the controls.

### RNA extraction and virus detection

Total RNA was isolated from leaf tissue of infected and healthy plants using a FastPure Plant Total RNA Isolation Kit (Vazyme, Nanjing, China), and 600 ng of total RNA was reverse transcribed into cDNA with the PrimeScript™ II 1st Strand cDNA Synthesis Kit (Takara Bio Inc., Dalian, China). Reverse transcription PCR (RT-PCR) was used to specifically detect CVB, CVR, CMV, TAV, TMV, PVY, CSVd, and CChMVd, as described previously [8, 9]. The primers used for virus detection are listed in Additional file 1.

### NGS, sequence assembling, and viral genome amplification

Total RNA was extracted from infected leaves using TRIzol reagent (Invitrogen, CA, USA) and the quantity and quality of total RNA were assessed using a NanoDrop ND-2000 spectrophotometer (NanoDrop Technologies, DE, USA). The cDNA library was constructed as described by Liu et al. [22] and Zhang et al. [23]. Subsequently, NGS was performed on an Illumina Hiseq 4000 platform (LC-Bio Technologies Co., Ltd., Hangzhou, China). The resulting raw reads were trimmed of adaptor sequences and low-quality reads and then assembled using Trinity (v.2.4.0) [24]. The assembled contigs were subsequently screened against the NCBI database (https://blast.ncbi.nlm.nih.gov/Blast.cgi) using BLASTn and BLASTx searches with standard parameters, as described previously [22, 23]. For phylogenetic analysis, the genome sequences were aligned using the ClustalW alignment and the phylogenetic trees were further constructed using the MEGA 11 [25], employing a neighbor-joining method with layouts of maximum composite likelihood model and 1000 bootstrap replications, as described by Zhang et al. [23]. The complete viral genome of CVB-CN was obtained by RT-PCR and 5′- and 3′-RACE-PCR using a SMARTer RACE 5′/3′ Kit (Takara Bio Inc.) with CVB-specific primers (Additional file 1) according to the manufacturer′s instructions.

### Transcriptome sequencing and functional annotation

For transcriptome sequencing, total RNA was extracted from healthy (control) and virus-infected samples and the quantity and quality assessment were performed, as described in Sect. 2.3. Next, the purified mRNAs were fragmented in Illumina proprietary fragmentation buffer, and then reverse-transcribed into cDNA libraries using the Truseq mRNASeq Sample Preparation Kit (Illumina, CA, USA). Subsequently, the constructed libraries were sequenced using the Illumina Hiseq 4000 platform (LC-Bio Technologies Co., Ltd.) in a paired-end mode, as described previously [26–28]. After removing of the adaptor sequences and low-quality reads, the resulting clean reads were de novo assembled using Trinity (v.2.4.0) [24], as described previously [26, 29].

The assembled unigenes were annotated through the NCBI non-redundant protein database (NR) (http://www.ncbi.nlm.nih.gov/), gene ontology (GO) (http://www.geneontology.org), SwissProt (http://www.expasy.ch/sprot/), Kyoto Encyclopedia of Genes and Genomes (KEGG) (http://www.genome.jp/kegg/), and eggNOG (http://eggnogdb.embl.de/) databases using DIAMOND (v.0.7.12) [30] with a threshold E-value < 0.00001, as described previously [31].

### Differential gene expression screening and enrichment analyses

For differential gene expression analysis, the transcripts per million (TPM) [32] at the gene level were counted and normalized using Salmon (v.0.8.2) [33]. To determine the differences in gene expression between virus-infected and control plants, an absolute value of Log2 fold change (FC) ≥ 1.0, and a Benjamini–Hochberg adjusted *P* value (*Padj*) < 0.05 was employed as the significance threshold, as described previously [26, 34, 35]. GO and KEGG enrichment analyses were performed using an in-house Perl script, and a false discovery rate (FDR) < 0.05 was considered to be significantly enriched, as described previously [36, 37].

### VIGS analysis

To disrupt the ethylene pathway in *N. benthamiana*, the tobacco rattle virus (TRV)-mediated VIGS system [38] was used to silence 1-aminocyclopropane-1-carboxylic acid (ACC) synthase 8 (*ACS8*), ACC oxidase 3 (*ACO3*), and ethylene insensitive 3 (*EIN3*) genes, which play crucial roles in the ethylene pathway [39]. The VIGS constructs were produced as described previously [40] and the primers used in the construction are listed in Additional file 1. Agroinfiltration of *N. benthamiana* was performed as described by Zhong et al. [41], and coinoculation of TRV1 with TRV2:GFP was used as the control. The silencing efficiency of VIGS was determined as described previously [39, 40].

### PVX construct and agroinfiltration

To generate the PVX expression construct, the coding sequence of CRP of CVB-CN (CVB-CN CRP) was cloned into the ClaI–SalI sites of the PVX vector, pGR106 [42]; the primers used in the construction are listed in Additional file 1. After confirming the sequence, the resulting plasmid, PVX:CVB-CN CRP, was mobilized into *Agrobacterium* strain GV3101 by electroporation. When expressing CVB-CN CRP in *N. benthamiana*, the *Agrobacterium* culture was resuspended in an infiltration buffer to a final optical density at 600 nm (OD600) of 0.8, as described previously [40, 41].

### ACC extraction and measurement

At 9 days post inoculation (dpi) of PVX:CVB-CN CRP, 100 mg of systemically infected leaves of *N. benthamiana* were sampled. The fresh samples were ground with 1.0 mL 0.1 M phosphate buffer solution (pH 7.4), and then centrifuged (5000 × *g*, 4 °C, 2 × 10 min). The supernatant was used for the ACC measurement. ACC content was determined using a Plant ACC ELISA Kit (Qiyuan Biological Technology Co., Ltd, Shanghai, China) according to the manufacturer′s instructions. ACC content was recorded per gram fresh weight, and each measurement was performed in triplicate.

### Quantitative real-time PCR (qRT-PCR) analysis

Total RNA from different leaf samples was extracted using the FastPure Plant Total RNA Isolation Kit (Vazyme) and genomic DNA contamination was cleaned with DNase I, as described previously [40, 43]. cDNA synthesis and qRT-PCR were performed as described by Wang et al. [43]. For each candidate gene, PCR was performed twice for each biological replicate and relative mRNA expression levels were calculated using the 2^–ΔΔCt^ method [44] based on three independent biological replicates. *N. benthamiana UBC* (*NbUBC*) or *C. morifolium EF1α* (*CmEF1α*) was used as an internal control, as described previously [45, 46]. The primers used for qRT-PCR analysis are listed in Additional file 1.

### Statistical analysis

Differences in the mean values were analyzed using the DPS software (v.18.10) [47] and assessed by one-way analysis of variance (ANOVA), followed by the Tukey′s test at *P < 0.05 and **P < 0.01.

## Results

### Identification of CVB-CN from C. morifolium

**Fig. 1:**
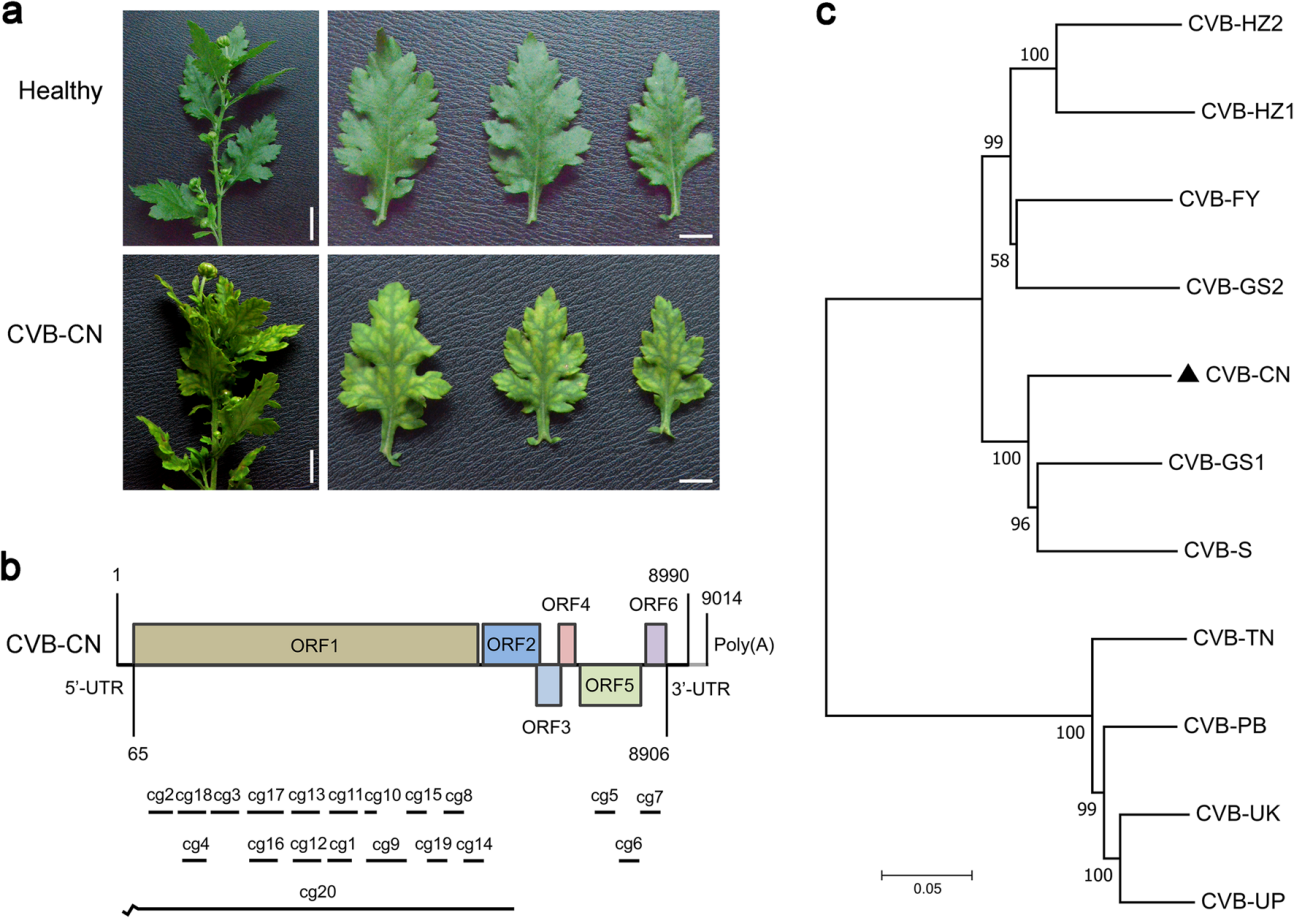
Symptoms, genome, and phylogenesis of the Chinese isolate of chrysanthemum virus B (CVB-CN). **a** Symptoms of CVB-CN on *Chrysanthemum morifolium* leaves. **b** Genome structure of CVB-CN. The putative open reading frames (ORFs) are indicated with different rectangles and 20 contigs assembled from virus-derived small RNAs are shown with black lines. **c** Evolutionary analysis of CVB-CN and the other 10 CVBs based on whole genome sequences. The trees were constructed with MEGA 11 using the neighbor-joining method with maximum composite likelihood model, and bootstrap values were estimated using 1000 replicates. The NCBI accession numbers of the other 10 CVB isolates with the whole genome sequences reported were: CVB-GS1 (MZ514908), CVB-S (AB245142), CVB-HZ2 (MW269553), CVB-HZ1 (MW269552), CVB-FY (MZ514910), CVB-GS2 (MZ514909), CVB-PB (AM493895), CVB-TN (AM765839), CVB-UK (AM765838), and CVB-UP (AM765837)

During a survey of viral diseases on October 13, 2018, chrysanthemum plants with severe leaf mottling and moderate vein clearing were observed in Tongxiang, Zhejiang Province, which is the most important chrysanthemum-growing area of Southeast China (Fig. 1a). These symptoms were similar to those caused by CVB, which has been reported in India [12, 13] and Japan [11]. To determine the causal agent(s) of these symptoms, total RNA was extracted from symptomatic leaves and subjected to RT-PCR analysis using primer pairs for the specific detection of CVB, CVR, CMV, TAV, TMV, PVY, CSVd, and CChMVd, which are epidemic in China [6, 8, 9]. As expected, a specific PCR band for CVB was obtained (Additional file 2), indicating that these samples might have been infected with CVB. To obtain the whole genome sequence of this CVB, a NGS analysis was performed as described previously [22, 23]. As expected, 20 contigs assembled from virus-derived small RNAs were specifically mapped to the genome of CVB from chrysanthemum (Fig. 1b and Additional file 3).

Next, the complete viral genome was obtained by RT-PCR and 5′- and 3′-RACE-PCR using CVB-specific primers (Additional file 1) designed on the basis of these contig sequences (Additional file 3). The assembled genome of this virus was 9014 nt in length (Fig. 1b and Additional file 4) and deposited in GenBank (https://www.ncbi.nlm.nih.gov/genbank/) as accession number MW691877. It had the highest nucleotide sequence identity (86.0%) to CVB-GS1 (GenBank: MZ514908) (Table 1) and contained a 64-nt-long 5′-untranslated region (UTR) (5′-UTR), 84-nt-long 3′-UTR, and 24-nt-long poly (A) tail (Fig. 1b). Its genome organization is typical of *Carlavirus*, containing six ORFs and encodes six putative viral proteins [11, 13, 21]. ORF1 (6285 nt, 65–6349) encodes a large putative replicase polyprotein (2094 aa, 237.1 kDa) that contains domains for viral methyltransferase (PF01660, aa 43–354), a *Carlavirus* endopeptidase (PF05379, aa 1095–1182), an RNA virus helicase (PF01443, aa 1269–1548), and an RNA-dependent RNA polymerase (PF00978, aa 1665–2082). ORFs 2–4 (1182 nt, 6383–7564) encode three TGB proteins (TGBp1, 2, and 3, respectively). ORF 5 encodes a CP, and ORF 6 (324 nt, 8583–8906) encodes a CRP [14, 17, 21]. Further phylogenetic analysis of this virus and of the other 10 CVB isolates, the whole genome sequences of which are reported, showed that this virus was closer to the CVB-GS1, with a whole-genome sequence identity of 86.0%, and was far from CVB-UP and CVB-UK, with whole-genome sequence identities of 70.0% and 71.0%, respectively (Fig. 1c and Table 1). Collectively, these results for this virus did not meet the molecular criteria for demarcation of species of *Carlavirus* [13, 48]. Thus, this virus should be considered a new CVB isolate and was designated as a Chinese isolate of CVB (CVB-CN).

**Table 1:** Nucleotide and amino acid sequence identities (%) of the Chinese isolate of chrysanthemum virus B (CVB-CN) and the other 10 CVB isolates

Viruses	Accession no.	Full length (nt^a^)	5′-UTR (nt^a^)	3′-UTR (nt^a^)	ORF1 (aa^b^)	ORF2 (aa^b^)	ORF3 (aa^b^)	ORF4 (aa^b^)	ORF5 (aa^b^)	ORF6 (aa^b^)
CVB-GS1	MZ514908	86.0	81.0	95.0	92.0	90.0	93.0	91.0	93.0	94.0
CVB-S	AB245142	85.0	98.0	94.0	90.0	90.0	90.0	88.0	93.0	95.0
CVB-HZ2	MW269553	83.0	92.0	95.0	88.0	86.0	91.0	91.0	91.0	96.0
CVB-HZ1	MW269552	81.0	97.0	93.0	87.0	87.0	91.0	81.0	92.0	90.0
CVB-FY	MZ514910	81.0	70.0	93.0	87.0	87.0	93.0	86.0	91.0	95.0
CVB-GS2	MZ514909	81.0	81.0	96.0	88.0	89.0	92.0	88.0	92.0	93.0
CVB-PB	AM493895	71.0	NA^c^	51.0	59.0	87.0	93.0	86.0	91.0	95.0
CVB-TN	AM765839	71.0	NA^c^	51.0	59.0	89.0	89.0	88.0	87.0	95.0
CVB-UK	AM765838	71.0	NA^c^	50.0	59.0	85.0	92.0	83.0	80.0	98.0
CVB-UP	AM765837	70.0	NA^c^	50.0	59.0	86.0	93.0	86.0	79.0	93.0

^a^*nt* Nucleotide

^b^*aa* amino acid

^c^*NA* not applicable

### Transcriptome sequencing of C. morifolium infected with CVB-CN

**Fig. 2:**
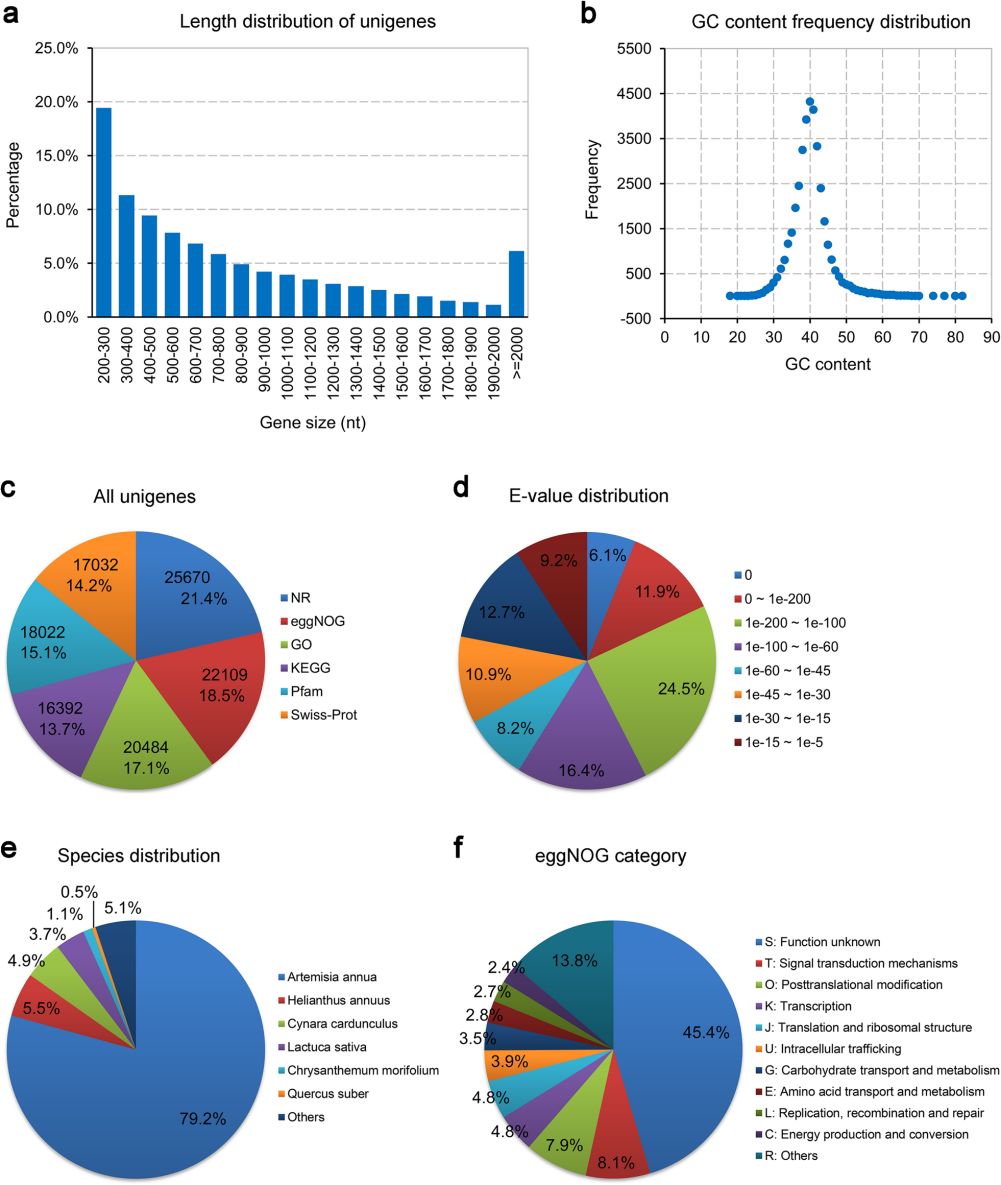
Transcriptome sequencing of leaves of *Chrysanthemum morifolium* following infection with the Chinese isolate of chrysanthemum virus B (CVB-CN). **a** The length distribution of all assembled unigenes. **b** The GC content frequency distribution of all assembled unigenes. **c** The number of unigenes annotated by different databases, including NR, eggNOG, GO, KEGG, Pfam, and Swiss-Prot. **d** E-value distribution of all NR-annotated unigenes. **e** Species distribution of all NR-annotated unigenes. **f** Category classification of all eggNOG-annotated unigenes

To investigate the DEGs of chrysanthemum in response to CVB-CN infection, a comprehensive transcriptome analysis of leaves of chrysanthemum uninfected (healthy) or infected with CVB-CN was performed using RNA sequencing. Six samples (three biological replicates from healthy controls and three biological replicates from CVB-CN-infected samples) were subjected to RNA-sequencing and approximately 11.78 Gb valid data were generated for each sample (Additional file 5). Subsequently, the clean reads gained from healthy and CVB-CN-infected leaf samples were de novo assembled using Trinity (v.2.4.0) [24], producing 37,112 unigenes (N50: 1188), with an average length of 627 bp (Additional file 6). As shown in Fig. 2a, there were 25,940 unigenes (69.9%) with lengths ≤ 1000 bp, 8900 unigenes (24.0%) in the range of 1000–2000 bp, and 2272 (6.1%) with lengths > 2000 bp in our transcriptome profiles. The GC content frequency distribution for all the unigenes is illustrated in Fig. 2b. As shown in Fig. 2c, 25,670 unigenes (69.2%) matched the protein sequences in the NR database, 22,109 (59.6%) in the eggNOG database, 20,484 (55.2%) in the GO database, 16,392 (44.2%) in the KEGG database, 18,022 (48.6%) in the Pfam database, and 17,032 (45.9%) in the Swiss-Prot database. The distribution of the E-values for the NR-annotated unigenes is illustrated in Fig. 2d. As shown in Fig. 2e, the largest number of *C. morifolium* homologous genes was found in *Artemisia annua*, a medicinal plant. For the eggNOG classification, a total of 22,109 unigenes were classified into 23 clusters of orthologous groups (COG) categories. The COG terms of “Signal transduction mechanisms”, “Posttranslational modification”, and “Transcription” made up the three largest categories except for the term of “Function unknown”. Interestingly, a similar number of unigenes belonged to the terms of “Signal transduction mechanisms” and “Posttranslational modification”, suggesting important roles played by these two categories in response to CVB-CN infection (Fig. 2f).

### Screening of significant DEGs (SDEGs) in C. morifolium responding to CVB-CN

**Fig. 3:**
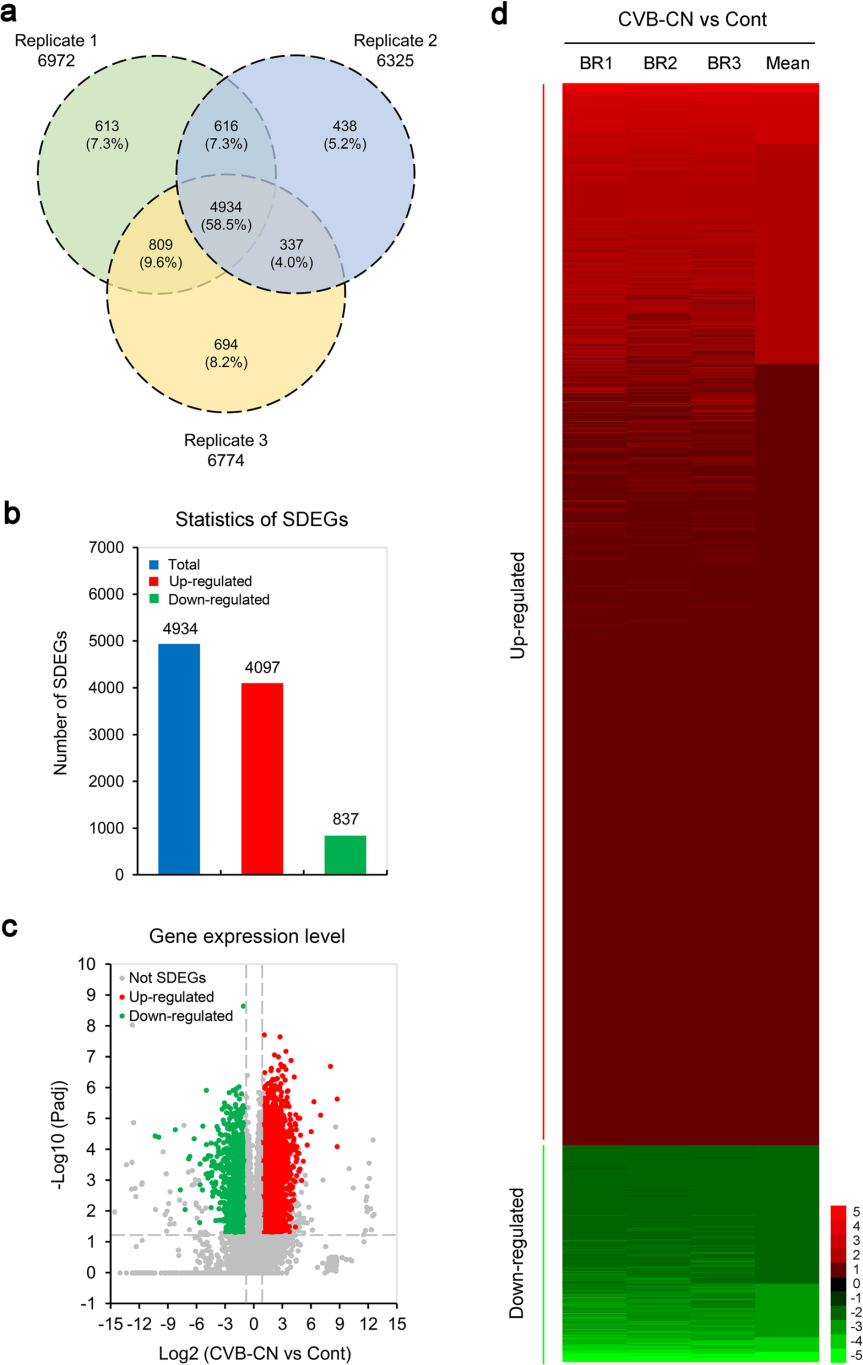
Screening of significant differentially expressed genes (SDEGs) in *Chrysanthemum morifolium* in response to the Chinese isolate of chrysanthemum virus B (CVB-CN). **a** Venn diagram analysis of SDEGs identified in *C. morifolium* following CVB-CN infection. **b** Number of the total, upregulated and downregulated SDEGs in *C. morifolium* following CVB-CN infection. **c** Volcano plot analysis of SDEGs identified in *C. morifolium* following CVB-CN infection. **d** Heatmap analysis of SDEGs identified in *C. morifolium* following CVB-CN infection

To determine the DEGs involved in the response of chrysanthemum to CVB-CN, TPM values were employed to measure the transcript abundance of each unigene. Compared with the control plants, 6972, 6325, and 6774 DEGs from biological replicates 1, 2, and 3, respectively, showed differential changes following CVB-CN infection, sharing a subset of 4934 DEGs in all three replicates (Fig. 3a). To increase the reliability of the transcriptome data, the DEGs identified in all the three replicates were considered as SDEGs in the present study. As a result, 4934 SDEGs (Additional file 7) were identified in the leaves of *C. morifolium* following CVB-CN infection, including 4097 upregulated and 837 downregulated genes. Interestingly, the number of upregulated SDEGs was nearly five times as much as that of the downregulated SDEGs in *C. morifolium* following CVB-CN infection (Fig. 3b). Significance analyses of the SDEGs between CVB-CN-infected and control plants were also performed using a volcano plot (Fig. 3c) and a heatmap (Fig. 3d). These results suggest substantial changes in the gene abundance in *C. morifolium* caused by CVB-CN at the transcriptional level.

**Fig. 4:**
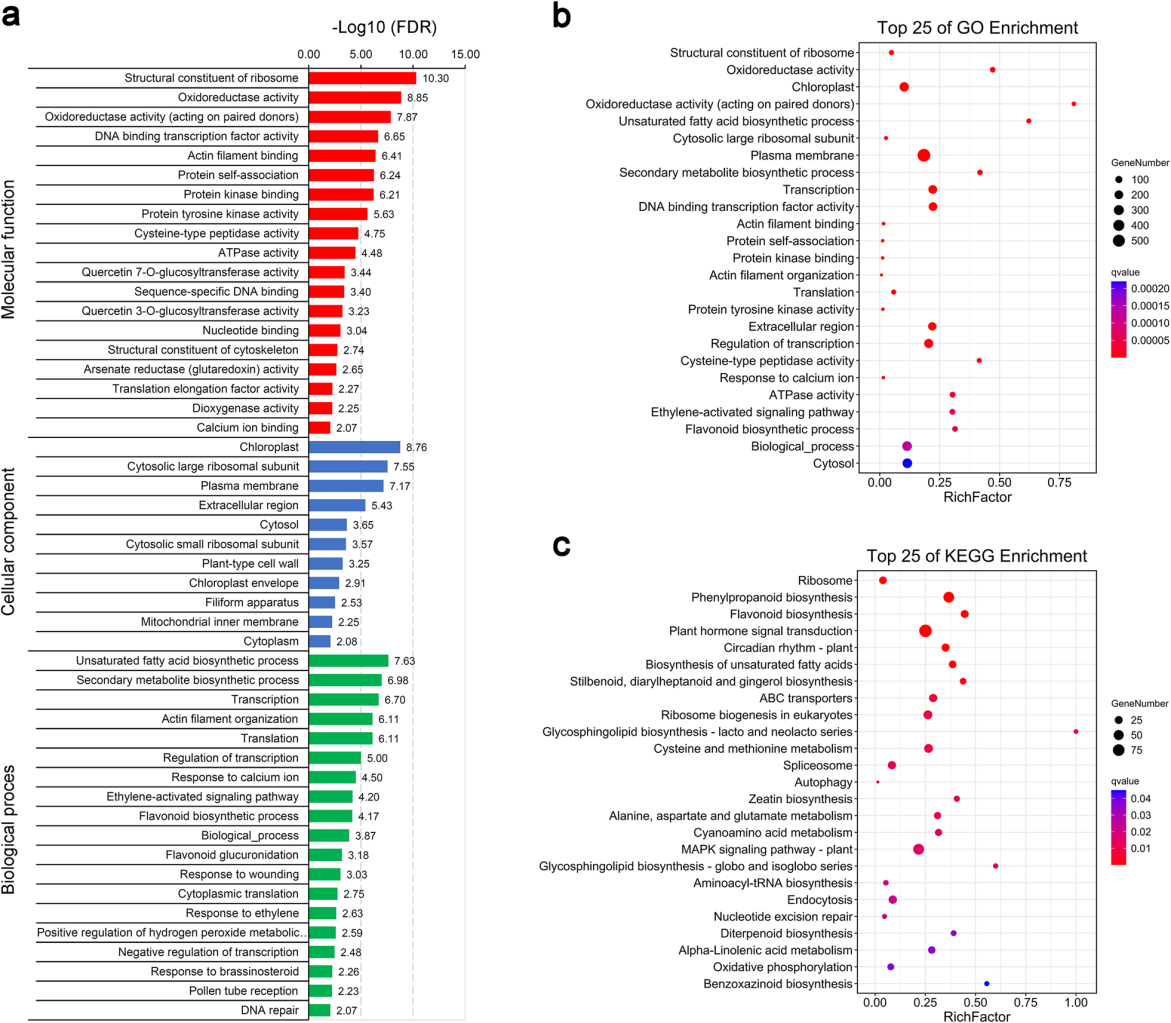
Gene ontology (GO) and Kyoto Encyclopedia of Genes and Genomes (KEGG) enrichment analyses of the significant differentially expressed genes (SDEGs) in *Chrysanthemum morifolium* in response to CVB-CN. **a** GO enrichment analysis of the SDEGs. **b** and **c** GO and KEGG enrichment analyses of the SDEGs depicted using bubble diagrams. The top 25 enriched terms are shown

To obtain more information about the potential functions of these CVB-CN-responsive SDEGs in chrysanthemum, GO enrichment analysis was carried out as described previously [36, 37]. Among these SDEGs, 87 GO terms implicated in molecular function, cellular component, and biological process categories were significantly enriched using a FDR < 0.05 (Fig. 4a and Additional file 8). As shown in Fig. 4b, the GO terms of “Structural constituent of ribosome”, “Oxidoreductase activity”, “Chloroplast”, “Oxidoreductase activity (acting on paired donors)”, “Unsaturated fatty acid biosynthetic process”, “Cytosolic large ribosomal subunit”, “Plasma membrane”, “Secondary metabolite biosynthetic process”, “Transcription”, and “DNA binding transcription factor activity” made up the ten largest GO terms of chrysanthemum in response to CVB-CN infection. Moreover, to determine the different metabolic pathways activated in response to CVB-CN infection, the SDEGs were assigned to the canonical pathways in the KEGG database, as described previously [\* MERGEFORMAT 26]. As a result, 2401 of these SDEGs could be assigned to 225 predicted metabolic pathways, of which 43 pathways were highly enriched (*P* < 0.05). After FDR correction, 25 pathways including “Ribosome”, “Phenylpropanoid biosynthesis”, “Flavonoid biosynthesis”, and “Plant hormone signal transduction” were significantly enriched (FDR < 0.05) (Fig. 4c and Additional file 9), indicating that these metabolic pathways might play potential roles in response to CVB-CN in chrysanthemum. These findings agree with those of previous studies that showed that viral infections can cause comprehensive rearrangements of cellular components and metabolism of host plants [26, 49, 50].

### Differential expression of transcription- and transcription regulation-related genes

**Fig. 5:**
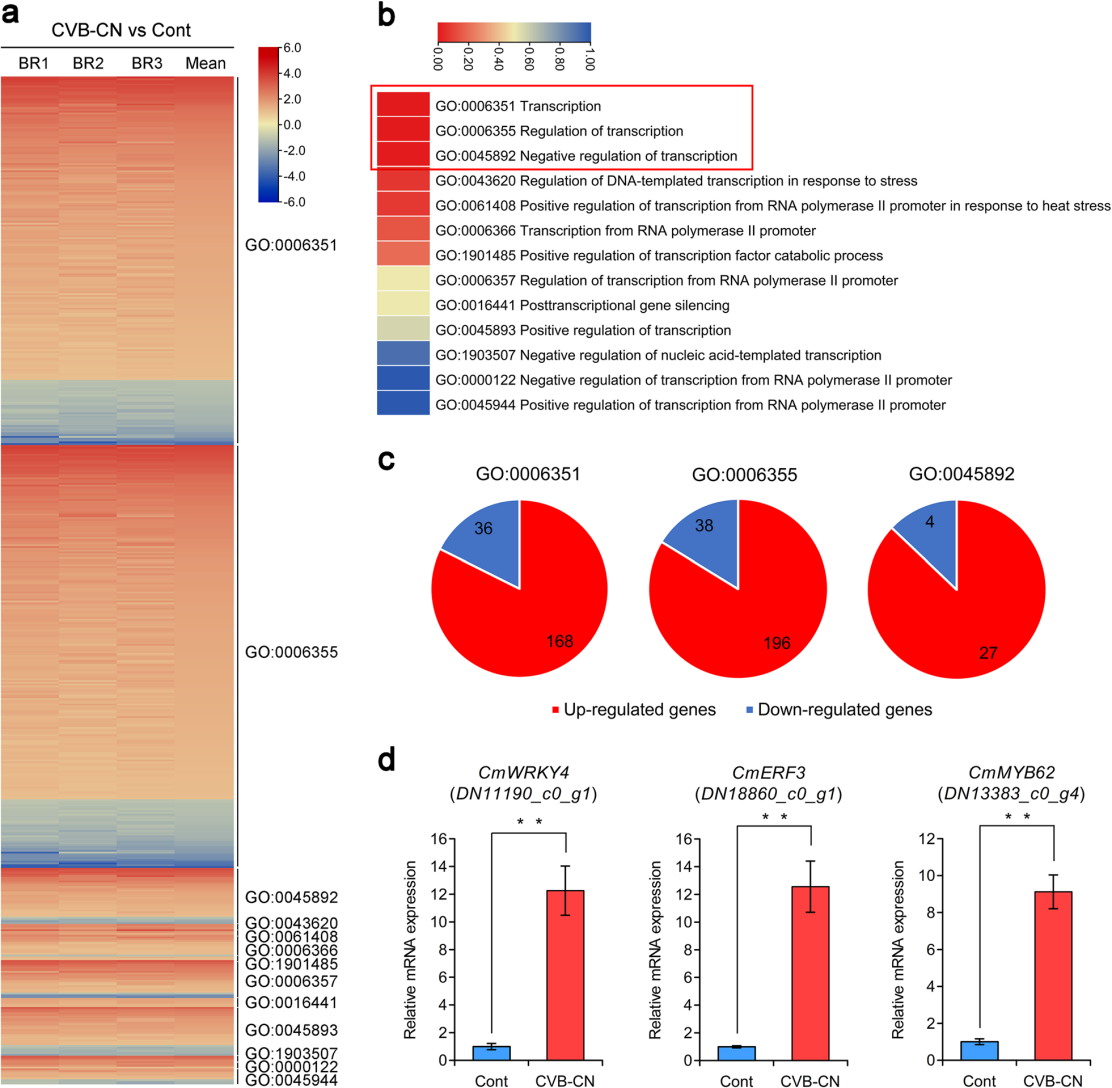
Analysis of the transcription- and transcription regulation-related genes in *Chrysanthemum morifolium* infected with the Chinese isolate of chrysanthemum virus B (CVB-CN). **a** Expression profiles of the genes related to transcription and transcription regulation are shown using the heatmap. All the transcription- and transcription regulation-related genes were assigned to 13 gene ontology (GO) terms. **b** Significance analysis of 13 transcription- and transcription regulation-related GO terms following CVB-CN infection. The red box indicates significant GO terms. **c** The number of upregulated and downregulated genes that were grouped into GO:0006351, GO:0006355, and GO:0045892. **d** Relative expression levels of three representative defense-related transcription factor genes of *C. morifolium* following CVB-CN infection. The data are given as means ± standard deviation of three biological replicates. Significant differences in expression are indicated with asterisks: (*) *P* < 0.05 or (**) *P* < 0.01; Tukey′s test

It has been shown that comprehensive transcriptional regulatory networks and signaling processes mediated by various transcriptional regulators are involved in plant defense responses to viral infections [51–53]. In the present study, 290 SDEGs associated with in transcription and transcription regulation were identified, of which 246 were upregulated in chrysanthemum during CVB-CN infection (Additional file 10). Further GO enrichment analysis showed that these 290 SDEGs were mainly categorized into 13 GO terms and the GO terms of “Transcription (GO:0006351)”, “Regulation of transcription (GO:0006355)”, and “Negative regulation of transcription (GO:0045892)” were significantly enriched using a FDR < 0.05 during CVB-CN infection (Fig. 5a,b). Interestingly, most of the SDEGs involved in these GO terms were up-regulated (Fig. 5c), suggesting that complex transcriptional reprogramming occurs in chrysanthemum during CVB-CN infection. This finding is in agreement with previous studies wherein it was observed that a specific set of transcription- and transcription regulation-associated genes accumulates and is enriched in *N. benthamiana* during viral infection [53, 54].

Transcription factors are essential components of plant defense signaling and resistance mechanisms through sequence-specific interactions with cis-regulatory elements in the promoters of target host defense response-related genes in response to pathogen infection [55]. Among the upregulated SDEGs, 160 were annotated as transcription factors that covered several families involved in plant defense responses (Additional file 10). In pepper, CaWRKYa, CaWRKYb, and CaWRKYd were reported to regulate innate immunity and/or hypersensitive responses during TMV infection and play positive roles in plant defense against TMV [56–58]. In *N. benthamiana*, NbWRKY40 was demonstrated to positively regulate the defense response to tomato mosaic virus (ToMV) via salicylic acid signaling, and overexpression of NbWRKY40 could inhibit ToMV infection and movement [59]. In our transcriptome data, compared with the control plants, 24 WRKY-related transcription factor genes were upregulated in *C. morifolium* infected with CVB-CN (Additional file 10). This suggests that WRKY-related transcription factors are also important regulators of the defense responses of chrysanthemum to CVB-CN infection. Furthermore, 22 AP2/ERF-related transcription factor genes were found to be significantly induced by CVB-CN in *C. morifolium* (Additional file 10). This finding aligns with previous studies showing that AP2/ERF-related transcription factors are key transcriptional regulators and are crucial for host defense responses against viral infection in tobacco [60] and tomato [61]. Moreover, other defense-related transcription factors, including MYB, bHLH, NAC, and bZIP families, which are involved in plant defense responses to viral infection [62–64], were also identified in our study (Additional file 10). To validate the expression levels of defense-related transcription factor genes under CVB-CN infection, three representative genes, including the *transcription factor WRKY4* (*WRKY4*) (*DN11190_c0_g1*), an *ethylene response DNA binding factor 3* (*ERF3*) (*DN18860_c0_g1*), and a *MYB domain protein 62* (*MYB62*) (*DN13383_c0_g4*), were selected for qRT-PCR analysis. qRT-PCR showed increased expression levels following CVB-CN infection, consistent with the RNA-sequencing data (Fig. 5d). Taken together, these results suggest that transcriptional reprogramming and signaling regulated by transcription factors were established during CVB-CN infection, although the exact roles of these defense-related transcription factors need to be further examined in the future.

### Differential expression of ethylene pathway-related genes

**Fig. 6:**
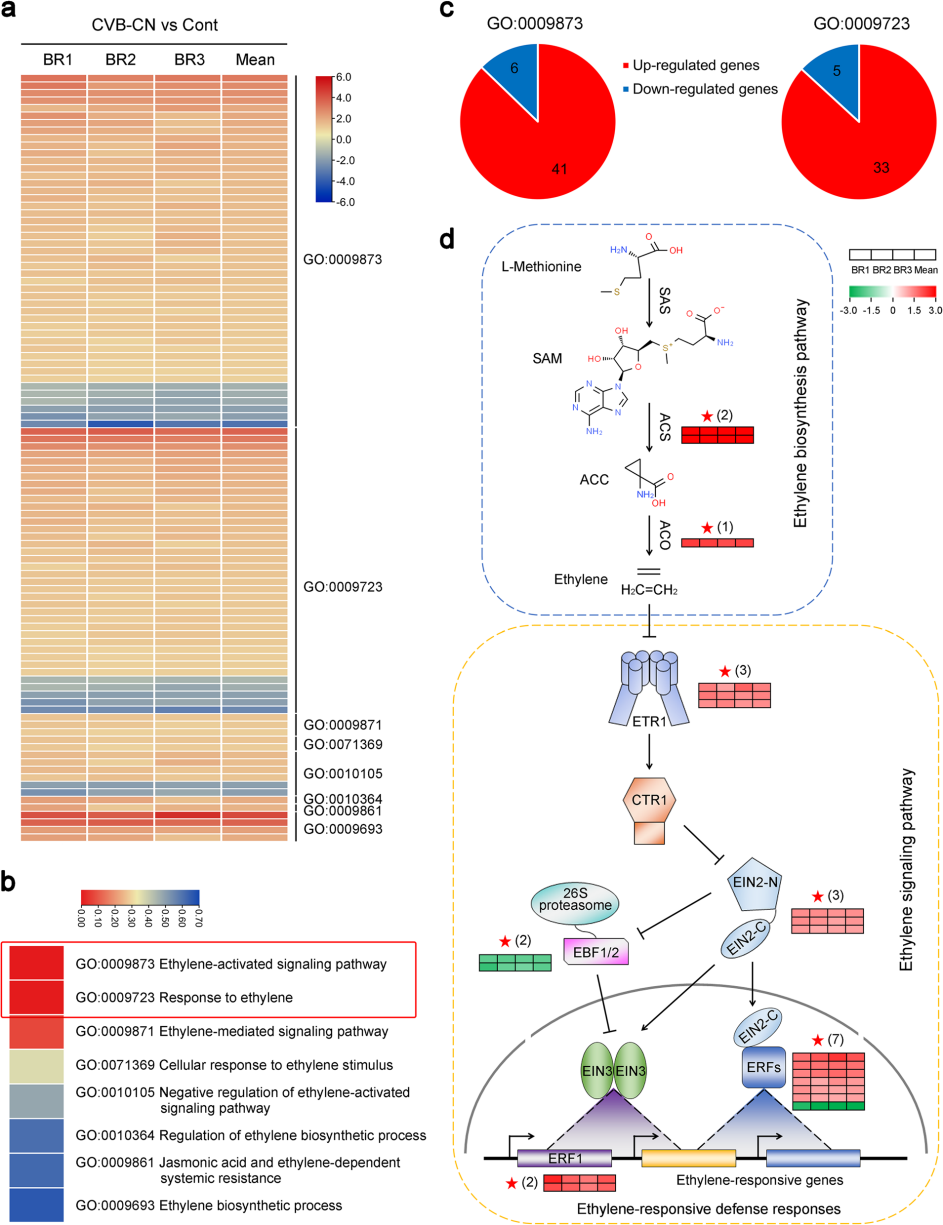
Expression of significant differentially expressed genes (SDEGs) related to ethylene biosynthesis and signaling pathway in *Chrysanthemum morifolium* infected with the Chinese isolate of chrysanthemum virus B (CVB-CN). **a** Expression profiles of the SDEGs related to ethylene biosynthesis and signaling pathway are illustrated using a heatmap. All the ethylene-related SDEGs were grouped into eight gene ontology (GO) terms. **b** Significance analysis of the eight ethylene-related GO terms under infection with the Chinese isolate of chrysanthemum virus B (CVB-CN). The red box shows significant GO terms. **c** The number of upregulated genes and downregulated SDEGs assigned to GO:0009873 or GO:0009723 terms. Red indicates upregulated genes and blue indicates downregulated genes. **d** A schematic representation of the SDEGs implicated in the ethylene pathway established based on the Kyoto Encyclopedia of Genes and Genomes (KEGG) database and previous studies on the model plant species *Arabidopsis*. Pentagrams () indicate the SDEGs identified during CVB-CN infection. Heat maps represent Log2 ratios of (CVB-CN/Cont) from three biological replicates. Arrows indicate activation of the pathway, and T-bars indicate the repression. SAM, *S*-adenosylmethionine; SAS, SAM synthase; ACC, 1-aminocyclopropane-1 -carboxylic acid; ACS, ACC synthase; ACO, ACC oxidase; ETR1, ethylene response 1; CTR1, constitutive triple response 1; EIN2, ethylene insensitive 2; EIN3, ethylene insensitive 3; EBF1/2, EIN3 binding F-box 1/2; ERF1, ethylene response factor 1; ERFs, ethylene-responsive transcription factors

Increasing evidence shows that ethylene is one of the key modulators of plant responses to viral infections [60, 65, 66]. In the present study, we compared the expression of the ethylene biosynthesis- and signaling pathway-related genes of *C. morifolium* in response to CVB-CN infection based on their GO terms. In total, 102 SDEGs (Additional file 11) were clustered into eight GO terms, including GO:0009873, GO:0009723, GO:0009871, GO:0071369, GO:0010105, GO:0010364, GO:0009861, and GO:0009693, which were identified as ethylene pathway-related genes (Fig. 6a). Among these GO terms, “Ethylene-activated signaling pathway” (GO:0009873, FDR = 0.0001) and “Response to ethylene” (GO:0009723, FDR = 0.0023) showed significant differences in *C. morifolium* in response to CVB-CN infection (Fig. 6b). GO:0009873 contained the largest number of ethylene pathway-related genes, including 41 upregulated and 6 downregulated SDEGs. In addition, GO:0009723 contained the second largest number of ethylene pathway-related genes, including 33 upregulated and 5 downregulated SDEGs (Fig. 6c). Next these ethylene pathway-related SDEGs were further mapped to the ethylene pathway established according to the KEGG database and previous studies on the model plant species *Arabidopsis* [67–69]. A diagram summarizing the ethylene pathway is shown in Fig. 6d. Two *ACS* genes (*DN19186_c0_g1* and *DN10045_c0_g1*), an *ACO* gene (*DN13216_c1_g1*), three *ethylene response 1* (*ETR1*) genes (*DN19772_c1_g1*, *DN21376_c0_g2*, and *DN13968_c2_g1*), three *EIN2* genes (*DN22236_c0_g1*, *DN20898_c0_g1*, and *DN20898_c0_g2*), two *EIN3 binding F-box 1/2* (*EBF1/2*) genes (*DN27110_c0_g2* and *DN13843_c0_g1*), two *ethylene response factor 1* (*ERF1*) genes (*DN9700_c0_g1* and *DN17171_c0_g1*), and seven *ethylene-responsive transcription factor* (*ERF*) genes (*DN22148_c1_g2*, *DN15303_c0_g4*, *DN21952_c1_g6*, *DN26663_c2_g1*, *DN23380_c0_g1*, *DN11554_c0_g1*, and *DN9752_c0_g1*) were implicated in ethylene biosynthesis- and signaling pathway and most of them significantly increased in their transcript abundances *C. morifolium* during infection with the CVB-CN (Fig. 6d and Additional file 12). Notably, the upregulated ethylene pathway-related SDEGs participated in several essential steps of ethylene biosynthesis and signaling. For example, in *N. tabacum*, overexpression of *NtERF5* can increase host resistance to TMV infection [60]. Likewise, in *N. benthamiana*, overexpression of *NbMYB4L*, an ethylene-induced transcription factor, also results in increased resistance to TMV [66]. Recently, *CaERF1A* was reported to be strongly induced by P0 of TMV (TMV-P0) and knockdown of *CaERF1A* negatively affected the TMV-P0-mediated hypersensitive response in hot pepper [70]. Collectively, these results suggest that the ethylene pathway may have a crucial function in the defense responses of chrysanthemum against CVB-CN infection.

### Disruption of the ethylene pathway enhances the CVB-CN CRP-induced hypersensitive response

**Fig. 7:**
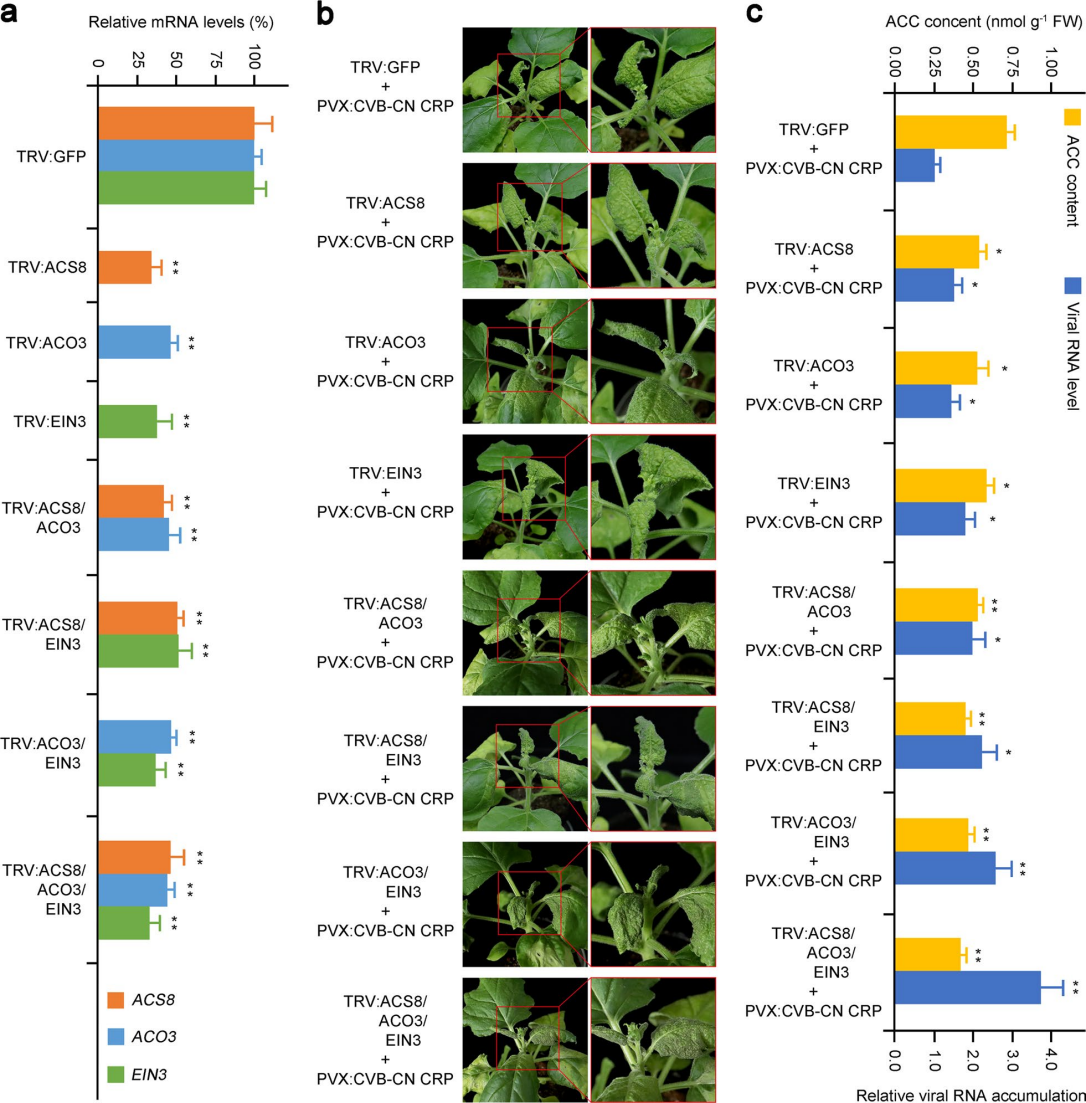
Disruption of the ethylene pathway in *Nicotiana benthamiana* makes it susceptible to infection with the cysteine-rich protein of the Chinese isolate of chrysanthemum virus B (CVB-CN CRP). **a** Silencing efficiency of virus-induced gene silencing (VIGS) assays was assessed by qRT-PCR at 10 days post inoculation (dpi). The data are given as means ± standard deviation of three biological replicates. The values represent relative mRNA levels against control groups (TRV:GFP-infiltrated *N. benthamiana* seedlings), values of which are set to 100%. Significant differences in expression are indicated with asterisks: (*) *P* < 0.05 or (**) *P* < 0.01; Tukey′s test. **b** Disease symptoms caused by PVX:CVB-CN CRP in the ethylene pathway impaired *N. benthamiana* seedlings at 9 dpi. TRV:GFP plants-inoculated with PVX:CRV-CN CRP were used as the control. **c** 1-Aminocyclopropane-1-carboxylic acid (ACC) content and relative viral RNA accumulation in the ethylene pathway-impaired *N. benthamiana* seedlings, as described in **b**. The data are given as means ± standard deviation of three biological replicates. The values represent ACC content or relative viral RNA levels against control groups (TRV:GFP-infiltrated *N. benthamiana* seedlings infected with PVX:CVB-CN CRP). Significant differences in expression are indicated with asterisks: (*) *P* < 0.05 or (**) *P* < 0.01; Tukey′s test

Next, we investigated whether the ethylene pathway plays a role in the defense responses of plants to CVB-CN. In the genus *Carlavirus*, it has been well demonstrated that CVB CRP is a symptom determinant and a viral suppressor of RNA silencing, which can induce a hypersensitive response in *N. tabacum* and *N. benthamiana* plants when expressed from a PVX vector [14, 17, 18]. Therefore, we examined the precise role of the ethylene pathway in plant defense responses in *N. benthamiana* using VIGS and PVX-expressing CVB-CN CRP. Based on our RNA-sequencing data, three key genes in the ethylene pathway, *ACS8*, *ACO3*, and *EIN3*, were selected and silenced individually or in combination with each other by TRV-mediated VIGS, as described previously [39]. At 10 dpi, the expression level of each gene was approximately 30–50% in TRV:GFP-infiltrated plants (Fig. 7a). PVX:CVB-CN CRP was then inoculated onto silenced plants using the agroinfiltration method, as described previously [17]. Compared with the TRV:GFP-infiltrated plants, more severe downward leaf curling and hypersensitive responses were observed on the systemic leaves of silenced seedlings at 9 dpi of PVX:CVB-CN CRP, especially in seedlings in which two or three ethylene pathway components were silenced (Fig. 7b). These results indicate that plants were more susceptible to CVB-CN CRP when the ethylene pathway was suppressed. For further confirmation, we determined the content of the precursor of ethylene, ACC, and viral CRP mRNA accumulation in the visible systemic leaves of *N. benthamiana* seedlings agroinfiltrated with PVX:CVB-CN CRP. The ACC content was considerably lower in silenced seedlings than in control plants (Fig. 7c). Notably, seedlings with silenced ethylene pathway components, especially those in which two or three of the ethylene pathway components were silenced, had higher levels of viral CRP mRNA accumulation than the control plants (Fig. 7c). Taken together, these results suggest that suppression of the ethylene pathway increased the susceptibility of *N. benthamiana* to CVB-CN CRP during virus–plant interactions.

## Discussion

In the past two decades, the NGS technology has been frequently used to assist in the discovery of new virus species [71–74]. In this study, the NGS analysis of the *C. morifolium* leaves demonstrated the presence of a virus related to the genus *Carlavirus* within the family *Betaflexiviridae* [11, 13]. Furthermore, the complete viral genome, which is 9014 nt in length, was obtained using RT-PCR and 5′- and 3′-RACE-PCR (Fig. 1b). Based on the bioinformatics analysis of the genomic features and phylogeny, the CVB-CN was determined to be a Chinese isolate of CVB that was closer to CVB-GS1 (Fig. 1c). Subsequently, comparative transcriptomic analysis of leaves was carried out to explore the mechanisms underlying the response of chrysanthemum to CVB-CN.

In the past decade, the availability of transcriptome data from host plants in response to RNA viruses has dramatically expanded. For example, data sets from the leaves of *N. tabacum* systemically infected with the M strain of CMV (M-CMV) with 8513 DEGs were generated by Lu et al. [75]; from the leaves of *D. grandiflorum* in response to CMV, tomato spotted wilt virus (TSWV), and PVX with 124 DEGs were produced by Choi et al. [76]; from the leaves of *L. regale* in response to CMV with 1346 DEGs were reported by Sun et al. [64]; from the leaves of *P. vulgaris* in response to soybean mosaic virus with 3548 DEGs were identified by Zhang et al. [77]; and from stems of *C. annuum* in response to three tobacco etch virus strains HAT, Mex21, and N having 24, 1190, and 4010 DEGs, respectively, were published by Murphy et al. [78]. However, to the best of our knowledge, transcriptome data on the mechanisms underlying the response of chrysanthemum to CVB are still not available.

In this study, the RNA sequencing-based comparative transcriptomic analysis of CVB-CN-infected and control *C. morifolium* leaves was carried out to further elucidate the mechanisms underlying the response of chrysanthemum to CVB-CN infection. A total of 4934 SDEGs, which contained 4097 upregulated and 837 downregulated genes, were screened in *C. morifolium* leaves following CVB-CN infection using three biological replicates (Fig. 3). This result indicates that a large quantity of transcriptional changes occurred during CVB-CN infection. This finding corroborates with those of previous studies wherein it was observed that host plants extensively alter their gene expression in response to viral infections [49, 75, 78]. GO enrichment analysis revealed that the CVB-CN responsive SDEGs were predominantly involved in the “Unsaturated fatty acid biosynthetic process”, “Secondary metabolite biosynthetic process”, “Transcription”, “Actin filament organization”, “Translation”, and “Regulation of transcription” in the biological process category (Fig. 4a,b), suggesting that these GO terms might be involved in the responses of chrysanthemum to CVB-CN. Among the top 25 enriched KEGG pathways, “Ribosome”, “Phenylpropanoid biosynthesis”, “Flavonoid biosynthesis”, and “Plant hormone signal transduction” were the four most significantly changed pathways in chrysanthemum in response to CVB-CN (Fig. 4c), indicating that the enhancement of these pathways might conduce to chrysanthemum responses and associated defense mechanisms to CVB-CN. These findings are in line with previous studies which showed that host metabolism, transcription, translation, and plant hormone signal transduction are frequently implicated in the defense responses of plants to viral infections [49–52, 55, 76, 77].

Besides its roles in plant development, fruit ripening, and organ senescence, ethylene is also a crucial modulator of plant responses to abiotic and biotic stimuli, including viral infections [65, 66, 79, 80]. Ethylene has been shown to modulate host defenses against viral infections in both positive and negative manners [39, 66, 80, 81]. For example, in *N. tabacum*, ethylene pathway has a crucial role in systemic resistance to Chilli veinal mottle virus (ChiVMV), and silencing of the ethylene biosynthetic and signaling components accelerate ChiVMV-induced cell death [81]. More recently, it was shown that ethylene can regulate the *NbMYB4L* gene to mediate resistance against TMV, and overexpression of *NbMYB4L* induces significant resistance to TMV in *N. benthamiana* [66]. In contrast, Zhao et al. [80] reported that ethylene plays a negative role in rice resistance to rice dwarf virus (RDV), and promotion of ethylene production results in elevated susceptibility to RDV. In *N. benthamiana*, the ethylene pathway had been reported to mediate the susceptibility to turnip mosaic virus (TuMV) and exogenous application of the ethylene precursor, ACC, enhances TuMV accumulation in infected plants [39]. Thus, ethylene has a complex role in the defenses of plants to viruses. In the present study, based on the KEGG database and previous studies on the model plant species *Arabidopsis*, 20 SDEGs were directly mapped to the ethylene biosynthesis- and signaling pathway (Fig. 6d). Interestingly, 17 SDEGs of them, which covered not only the ethylene biosynthesis pathway but also the ethylene signaling pathway, were upregulated in *C. morifolium* during infection with the CVB-CN (Fig. 6d and Additional file 12). It is important to notice that three *CTR1* genes were also upregulated following the CVB-CN infection (Fig. 6d and Additional file 12). We speculated that CTR1, a negative regulator of ethylene signaling, was were upregulated probably to reduce the damage caused by the excessive accumulation of virus-induced ethylene to normal plant growth and development, because that silencing of *CTR1* in tomato can enhance the upregulation of a set of defense-related genes and increase host resistance against tomato leaf curl virus infection [82]. To further investigate how the ethylene pathway is involved in plant resistance to CVB-CN, we examined the precise role of this pathway in *N. benthamiana* using VIGS and PVX-expressing CVB-CN CRP. Compared with the TRV:GFP-infiltrated plants, *N. benthamiana* plants with silenced ethylene pathway components displayed more severe downward leaf curling and hypersensitive response and had higher accumulation of CRP mRNA (Fig. 7). These results suggest that the ethylene pathway plays an essential role in plant resistance to CVB-CN. This finding will increase our understanding of the role of ethylene in plant defense responses and its associated mechanisms.

## Conclusions

In this study, the complete genome sequence of a Chinese isolate of CVB from its natural host was obtained using NGS and RT-PCR. Although genomic sequences of CVBs from several countries are known, more strategic and potential defense mechanisms employed by host plants remain to be elucidated. Comparative transcriptomic analysis revealed a variety of substantial transcriptional changes in chrysanthemum in response to CVB-CN infection. Bioinformatics analyses showed that ethylene pathway-related genes are significantly upregulated following CVB-CN infection. The potential role of the ethylene pathway in plants′ response to CVB-CN was analyzed using VIGS and PVX-expressing CVB-CN CRP and it was found that this pathway plays a positive role. These findings demonstrate that the combination of NGS, transcriptomics, and VIGS analyses is helpful in exploring the defense responses of plants to viral infections. It also provided important insights into the potential defense mechanisms employed by chrysanthemum against *Carlavirus*.

## Supplementary Information

**Additional file 1: Table S1**. List of primers used in this study.

**Additional file 2: Fig. S1**. Reverse transcription PCR (RT-PCR) analysis of symptomatic Chrysanthemum morifolium samples.

**Additional file 3: Table S2**. Contigs mapped in the viral genome of the Chinese isolate of chrysanthemum virus B (CVB-CN).

**Additional file 4: Table S3**. Full-length genome sequence of the Chinese isolate of chrysanthemum virus B (CVB-CN).

**Additional file 5: Table S4**. Summary of RNA sequencing of leaves of Chrysanthemum morifolium, uninfected (healthy) or infected with the Chinese isolate of chrysanthemum virus B (CVB-CN).

**Additional file 6: Table S5**. Summary of transcripts and unigenes assembled using the Trinity software.

**Additional file 7: Table S6**. Significant differentially expressed genes (SDEGs) identified in leaves of Chrysanthemum morifolium during infection with the Chinese isolate of chrysanthemum virus B (CVB-CN).

**Additional file 8: Table S7**. Gene ontology (GO) enrichment analysis of the significant differentially expressed genes (SDEGs) of Chrysanthemum morifolium responding to infection with the Chinese isolate of chrysanthemum virus B (CVB-CN).

**Additional file 9: Table S8**. Kyoto Encyclopedia of Genes and Genomes (KEGG) enrichment analysis of the significant differentially expressed genes (SDEGs) of Chrysanthemum morifolium responding to infection with the Chinese isolate of chrysanthemum virus B (CVB-CN).

**Additional file 10: Table S9**. Significant differentially expressed genes (SDEGs) in leaves of Chrysanthemum morifolium implicated in transcription and transcription regulation during infection with the Chinese isolate of chrysanthemum virus B (CVB-CN).

**Additional file 11: Table S10**. Significant differentially expressed genes (SDEGs) in leaves of Chrysanthemum morifolium involved in ethylene pathway based on gene ontology (GO) analysis during infection with the Chinese isolate of chrysanthemum virus B (CVB-CN).

**Additional file 12: Table S11**. Significant differentially expressed genes (SDEGs) in Chrysanthemum morifolium following infection with the Chinese isolate of chrysanthemum virus B (CVB-CN) implicated in the ethylene pathway established according to the Kyoto Encyclopedia of Genes and Genomes (KEGG) database and previous studies on the model plant species Arabidopsis.

## Data Availability

All data generated or analysed during this study are included in this published article and its supplementary information files. The genome sequence of the Chinese isolate of chrysanthemum virus B (CVB-CN) is available at the GenBank (https://www.ncbi.nlm.nih.gov/genbank/).

## Abbreviations

ACC: 1-Aminocyclopropane-1-carboxylic acid
ACO3: ACC oxidase 3
ACS8: ACC synthase 8
CChMVd: Chrysanthemum chlorotic mottle viroid
ChiVMV: Chilli veinal mottle virus
CMV: Cucumber mosaic virus
COG: Clusters of orthologous groups
CP: Coat protein
CRP: Cysteine-rich protein
CSVd: Chrysanthemum stunt viroid
CVB: Chrysanthemum virus B
CVB-CN: Chinese isolate of CVB
CVR: Chrysanthemum virus R
dpi: Days post inoculation
EIN2: Ethylene insensitive 2
EIN3: Ethylene insensitive 3
ERF: Ethylene-responsive transcription factor
ERF1: Ethylene response factor 1
ETR1: Ethylene response 1
FC: Fold change
FDR: False discovery rate
GO: Gene ontology
KEGG: Kyoto Encyclopedia of Genes and Genomes
NGS: Next-generation sequencing
ORF: Open reading frame
PVX: Potato virus X
PVY: Potato virus Y
qRT-PCR: Quantitative real-time PCR
SDEG: Significant differentially expressed gene
TAV: Tomato aspermy virus
TGB: Triple gene block
TMV: Tobacco mosaic virus
TPM: Transcripts per million
TRV: Tobacco rattle virus
TuMV: Turnip mosaic virus
UTR: Untranslated region
VIGS: Virus-induced gene silencing
