# Supplementary material for: Integrated next-generation sequencing and comparative transcriptomic analysis of leaves provides novel insights into the ethylene pathway of Chrysanthemum morifolium in response to a Chinese isolate of chrysanthemum virus B
Source: Virol J. 2022 Nov 11;19:182. doi: 10.1186/s12985-022-01890-3 (PMC9650830; doi:10.1186/s12985-022-01890-3)
Supplement: Supplementary file 2 — Additional file 2: Fig. S1. Reverse transcription PCR (RT-PCR) analysis of symptomatic Chrysanthemum morifolium samples. [file 12985_2022_1890_MOESM2_ESM.pdf]

Figure S1

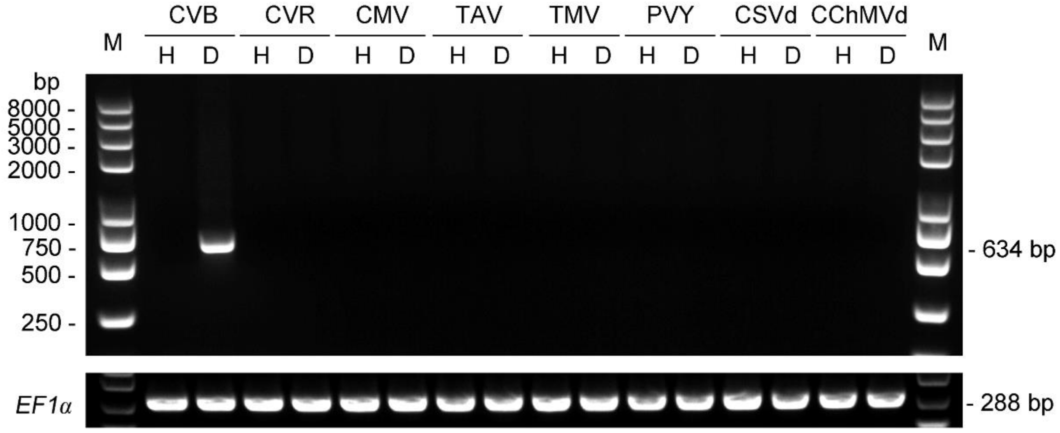

Figure S1. Reverse transcription PCR (RT-PCR) analysis of symptomatic *Chrysanthemum morifolium* samples. RT-PCR detection of the chrysanthemum virus B (CVB), chrysanthemum virus R (CVR), cucumber mosaic virus (CMV), tomato aspermy virus (TAV), tobacco mosaic virus (TMV), potato virus Y (PVY), chrysanthemum stunt viroid (CSVd), and chrysanthemum chlorotic mottle viroid (CChMVd). *C. morifolium EF1α* (*CmEF1α*) was used as an internal control. M: DNA marker; H: healthy leaf samples; D: diseased leaf samples.
